# Improving the ORS: Does Glutamine have a Role?

**Published:** 2007-09

**Authors:** Pradip K. Bardhan

**Affiliations:** Dhaka Hospital, ICDDR,B, GPO Box 128, Dhaka 1000, Bangladesh, bardhan@icddrb.org

Diarrhoea continues to be a leading cause of morbidity and mortality around the world, resulting in an estimated 1.5 billion episodes and 1.5 million to 2.2 million deaths annually among children aged less than five years (1,2). Oral rehydration therapy (ORT) effectively treats mild-to-moderate dehydration due to diarrhoea and, for the past three decades, has saved millions of lives, mostly in developing countries. A crucial aspect in this decrease in mortality due to diarrhoea has been the widespread acceptance of ORT for the prevention and treatment of dehydration associated with diarrhoeal illnesses (3). However, despite its remarkable success, the currently-recommended oral rehydration solution (ORS) does not significantly reduce stool volume or duration of diarrhoeal illness. Thus, the search continues for improved formulations of ORS capable of lessening the severity of diarrhoea.

The underlying physiologic principle of ORT is neat and simple—transport of sodium-coupled glucose across the small intestinal epithelial membrane remains largely intact in secretory diarrhoea and continues to stimulate absorption of water and electrolytes (4). Most attempts towards improving ORS have aimed at optimization of absorption of small intestinal fluid. Searching for an ORS capable of reducing diarrhoeal stool output, many clinical and basic scientists have investigated the role of other sodium co-transport substrates in addition to or instead of glucose. These included adding amino acids, e.g. glycine (5), alanine (6), and glutamine (7), and substitution of glucose by complex carbohydrates, e.g. maltodextrins (8), cooked rice-powder and other cereal powders (9,10). While the clinical trials using glycine, alanine, maltodextrins, and cereal-based ORSs conducted in the 1970s and 1980s showed promise, results of subsequent large trials and meta-analyses of results of previous trials found no advantage of most of these formulations when compared with the WHO-ORS, with the exception of rice-based ORS (11,12).

In the study published in this issue of the *Journal of Health, Population and Nutrition*, Guiterrez *et al*. compared the effects of a glutamine-containing glucose-free ORS with WHO-ORS in a double-blind randomized clinical trial among 147 dehydrated children with acute diarrhoea (13). Rotavirus was isolated from 62 of these children. The authors did not observe any significant difference between the two ORS groups in stool output during the first four hours, time taken to rehydrate successfully, volume of ORS needed to rehydrate, vomiting, and urinary output. This was independent of rotavirus-associated illness. The authors concluded that an L-glutamine-containing glucose-free ORS does not seem to offer any clinical benefit compared to the standard WHO-ORS in mild-to-moderately dehydrated children with acute non-cholera diarrhoea.

The amino acid glutamine is the fundamental respiratory fuel for the small intestine and has been classified as a conditional essential amino acid (14). Supplementation of glutamine has been shown to cause marked improvement in gastrointestinal structure and function after injury by chemotherapy, radiotherapy (15,16), or following prolonged parenteral nutrition (17). Glutamine also helps maintain the intestinal immunologic barrier, since it has been shown to increase the intestinal immunoglobulin A levels and to reduce bacterial translocation (18,19), and possibly the mechanism by which it reduces the incidence of bacteraemia and clinical infections (15,20). In addition, glutamine has also been postulated as a regulator of intracellular kinases, apoptosis, cell proliferation, redox status, and heat shock protein expression (21).

In addition to being the predominant intestinal epithelial fuel source, the fact that glutamine is able to function as a sodium co-transport substrate in an electrogenic manner and to stimulate electroneutral NaCl absorption (22) has generated interest in glutamine as a potentially-useful component in ORS. Experiments have clearly shown that glutamine is able to promote the absorption of sodium and water, even more effectively than glucose (23,24). In a perfused rabbit ileum model exposed to cholera toxin, a glutamine-based ORS caused a reduction of approximately 90% of cholera toxin-induced net sodium and water secretion (23); these observations are supported by other reports (24).

In a rat model of secretory diarrhoea induced by cholera toxin (25), glutamine was found to have a significant positive effect upon sodium absorption, but alanine-glutamine-based ORS had an even better effect in sodium absorption. However, it remains to be explained whether these effects of alanine-glutamine are due only to effective availability of glutamine or to a different mechanism. In adult cholera patients, *in vivo* intestinal perfusion experiments have shown that glutamine-containing ORS reduced electrolyte and water secretion, although no increased sodium or water absorption was observed when compared with the effects with ORS containing glucose alone (26). In dehydrated infants suffering from non-cholera acute diarrhoea, ORS containing glutamine was found to be well-tolerated and as effective as the standard WHO-ORS (7). In another study conducted among adults with cholera, use of glutamine-supplemented ORS was associated with a 25% reduction in stool volume in the first 24 hours and a 30% reduction in the total faecal loss (27). Glutamine has also been observed to stimulate sodium co-transport in experimental models of rotavirus-associated enteritis and enteropathogenic *Escherichia coli*-associated enteritis (28,29), although observations from other animal studies are not suggestive of a substantial effect of glutamine on acute diarrhoea (30).

Investigators, in efforts to enhance the effectiveness of ORS, have also pursued pathways other than optimization of small intestinal absorption. One approach has been to use the mechanism of colonic salvage of fluids unabsorbed in the small intestine. Short-chain fatty acids are the major colonic luminal anions and are able to enhance sodium absorption and inhibit chloride secretion in both normal and secreting colon (31,32). Short-chain fatty acids are formed in the colon by fermentation of unabsorbed starch and dietary fibres by the resident anaerobic bacterial flora. Several studies have examined the effects of addition of amylase-resistant starch and soluble dietary fibres to the standard ORS in diarrhoeal patients. In adult cholera patients, the group receiving amylase-resistant starch had had significantly reduced stool volumes and the duration of illness when compared with the group receiving the standard WHO-ORS (33). In non-cholera childhood diarrhoea, two studies reported that the addition of amylase-resistant starch or partially-hydrolyzed guar gum (as the short-chain fatty acid precursors) to the standard glucose-ORS significantly shortened the duration of diarrhoea (34,35), whereas another study using a mixture of non-digestible carbohydrates failed to observe any benefit (36). Taking a different approach, a group of investigators aimed at examining whether bioactive proteins found in breastmilk, when added to the standard ORS, yield any additional advantage. They observed that the addition of recombinant human lactoferrin and lysozyme to a rice-based ORS led to a significant decrease in the duration of diarrhoea in children with non-cholera acute diarrhoea (37). Yet, in another innovative approach—in an experimental study using *in vivo* small intestinal perfusion in rats exposed to cholera toxin or the 5-fluorouracil—ORS with partial incorporation of the ORS components into liposomes showed 40–50% increased absorption of water when compared with that from hypoosmolar WHO glucose-ORS (38). To assess the full potential of these various approaches in enhancing ORS, data from carefully-designed and conducted large-scale clinical trials require to be evaluated before deciding upon their effectiveness, practicality, applicability, and acceptability.

Among the various factors contributing towards the success of ORS, the critical factors include its low cost, simplicity, and ease of use. Based upon observations that using a reduced-osmolarity ORS caused fewer unscheduled intravenous infusions, lower stool volumes, and less vomiting when compared with the standard WHO-ORS (39), the WHO recently recommended a change in the composition of ORS to a low-sodium and low-glucose solution (40). This improvement in the effectiveness of ORS was achieved without compromising the cost, simplicity, or ease of use. In future, further enhancements of ORT will similarly depend upon a careful balance of clinical effectiveness, cost-effectiveness, safety, public-health benefits, and ease of use.
